# Isolation and Quantification of Mandelonitrile from *Arabidopsis thaliana* Using Gas Chromatography/Mass Spectrometry

**DOI:** 10.21769/BioProtoc.4700

**Published:** 2023-06-20

**Authors:** Ana Arnaiz, Lucas J. Vallejo-García, Saúl Vallejos, Isabel Diaz

**Affiliations:** 1 Universidad Politécnica de Madrid, Madrid, Spain; 2 Departamento de Química, Facultad de Ciencias, Universidad de Burgos, Burgos, Spain; 3 Centro de Biotecnología y Genómica de Plantas, Universidad Politécnica de Madrid—Instituto Nacional de Investigación y Tecnología Agraria y Alimentaria, Madrid, Spain; 4 Departamento de BiotecnologíaBiología Vegetal, Escuela Técnica Superior de Ingeniería Agronómica, Alimentaria y de Biosistemas, Universidad Politécnica de Madrid, Madrid, Spain

**Keywords:** Benzaldehyde cyanohydrin, Mandelonitrile, Hydroxynitrile lyase, *
Arabidopsis thaliana
*, Spider mite, Cyanogenic glycosides, Cyanohydrin

## Abstract

Mandelonitrile is a nitrogen-containing compound, considered an essential secondary metabolite. Chemically, it is a cyanohydrin derivative of benzaldehyde, with relevant functions in different physiological processes including defense against phytophagous arthropods. So far, procedures for detecting mandelonitrile have been effectively applied in cyanogenic plant species such as *Prunus* spp. Nevertheless, its presence in *Arabidopsis thaliana* , considered a non-cyanogenic species, has never been determined. Here, we report the development of an accurate protocol for mandelonitrile quantification in *A. thaliana* within the context of *A. thaliana* –spider mite interaction.

First, mandelonitrile was isolated from *Arabidopsis* rosettes using methanol; then, it was derivatized by silylation to enhance detection and, finally, it was quantified using gas chromatography–mass spectrometry. The selectivity and sensitivity of this method make it possible to detect low levels of mandelonitrile (LOD 3 ppm) in a plant species considered non-cyanogenic that, therefore, will have little to no cyanogenic compounds, using a small quantity of starting material (≥100 mg).

## Background

Cyanogenic glycosides (CNglcs) are secondary plant metabolites arisen from amino acids and found in over 3,000 plant species, including economically important crops as *Prunus* spp. *, Manihot* spp. *, Malus* spp., or *Sorghum* spp. ( Gleadow and Møller, 2014 ). CNglcs play crucial roles in different physiological processes such as germination, transport of essential nutrients, turnover processes, oxidative stress regulation, or defense responses ( Møller, 2010 ; Kadow et al., 2012; Flematti et al., 2013; Neilson et al., 2013 ). The metabolism of CNglcs is complex and derives in cyanohydrin production, which in turn can be converted into hydrogen cyanide and ketones or aldehydes in the presence of α-hydroxynitrile lyase enzymes (HNLs). Plant cyanohydrins have been demonstrated to act as direct defense molecules, with a highly toxic action against herbivores and pathogens ( Park et al., 2002 ; Beran et al., 2019 ). In addition, they can also work as signaling molecules, triggering the defense response ( Bernal-Vicente et al., 2020 ). However, some herbivores, mainly lepidopteran insects, have developed the ability to counteract or take advantage of these cyanogenic molecules, and alternatively may degrade or use these compounds for their benefit ( Zagrobelny and Møller, 2011 ; Zagrobelny et al., 2014).

**Figure 1.**
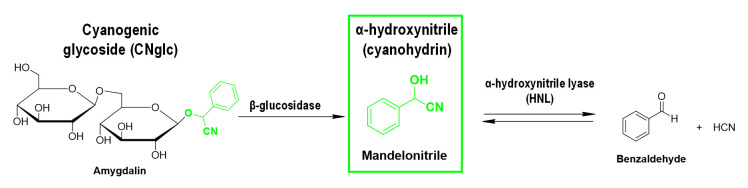
Example of a cyanogenesis pathway in plants with mandelonitrile as the central player. HCN: hydrogen cyanide.

The focus of this protocol is one of these CNglcs derivatives, mandelonitrile. Mandelonitrile is a cyanohydrin derived from CNglc metabolism, as prunasin or amygdalin among others ( Gleadow and Møller, 2014 ), that occurs naturally in cyanogenic plants. It is characterized by having hydroxyl and cyano groups attached to the same carbon atom ( Figure 1 ). Some works have reported the quantification of mandelonitrile in different cyanogenic plant species such as *Prunus cerasus* L. and *Aronia melanocarpa* Ell using high-performance liquid chromatography (HPLC) and gas chromatography–mass spectrometry (GC/MS) techniques, respectively ( Hirvi and Honkanen, 1985 ; Chandra and Nair, 1993 ).

Chassagne et al. (1996) detected amygdalin and its corresponding parental ion, benzaldehyde cyanohydrin (or mandelonitrile), in Passiflora fruits using GC/MS. More recently, mandelonitrile has been quantified in peach micro-propagated shoots by ultra-performance liquid chromatography (UPLC) coupled to a mass spectrophotometer ( Bernal-Vicente et al., 2020 ). However, as far as we know, mandelonitrile levels haven’t been determined in the model plant *A. thaliana* until now, maybe because it is considered a non-cyanogenic plant (CNglcs have not been detected in *A. thaliana* wild-type plants). Despite this, an alternative pathway to cyanohydrin production has recently been described—the 4-hydroxy-indole-3-carbonylnitrile (4-OH-ICN) route—as a rare metabolic re-invention leading to alternative cyanogenic compounds, to expand *A. thaliana* defenses and also contribute to the production of cyanohydrins ( Rajniak et al., 2015 ; Pastorczyk et al., 2020 ). In addition, an HNL has also been found in *A. thaliana* , and its encoding gene has been cloned and the recombinant protein has been purified and crystalized ( Andexer et al., 2007 ). Biochemical studies revealed that *Arabidopsis* HNL recognized a broad range of substrates, was enantioselective, and transformed aliphatic and aromatic aldehydes and/or ketones into the corresponding R-cyanohydrins, including mandelonitrile ( Andexer et al., 2007 ).

Therefore, the study of the relationship between mandelonitrile levels and the role of HNL in *Arabidopsis* has allowed the establishment of their defense role against the two-spotted spider mite *Tetranychus urticae* ( Arnaiz et al., 2022 ). Consequently, mandelonitrile can be considered a target molecule for the study of *Arabidopsis* –spider mite interaction in the plant defense process. This is why the development of a protocol to determine the levels of mandelonitrile in *A. thaliana* plants under controlled conditions and after spider mite infestation is of crucial interest. In addition, this protocol was established since it was not possible to adapt previous protocols already described for other plant species. In fact, no results were obtained using HPLC techniques and with the available quantity of infested *A. thaliana* sample as starting plant material, suggesting the need to develop a specific quantification procedure for such a non-cyanogenic species. GC/MS was selected for mandelonitrile detection as it allowed to detect the target molecule in standard samples. Moreover, the spectrum information related to mandelonitrile-derivatized found in the databases helped us to confirm our obtained results. Additionally, the derivatization process of mandelonitrile makes it a very volatile molecule and for that reason, it was better to use GC/MS than HPLC for analysis. Finally, it is likely that this protocol will work in other plant species by applying some modifications, e.g., quantity of starting material, solvent volume, or incubation and sonication times.

## Materials and reagents

**Biological material**

1. *
Arabidopsis thaliana* Columbia-0 (Col-0) ecotype seeds were purchased at the Arabidopsis Biological Resource Center
2. *
Tetranychus urticae* , London strain (Acari: Tetranychidae). Dr. Miodrag Grbic (UWO, Canada) provided the spider mite colony (see Note 1)

**Materials**

1. Microcentrifuge tube with safe lock 1.5 mL (Astik’s, catalog number: PCRP-015-500)
2. Pipette tips (Astik’s, catalog numbers: TIPP-1K0-10K, TIPP-200-10K, and TIPP-010-10K)
3. Peat moss (Klasmann-Deilmann, catalog number: L1070K0600)
4. Vermiculite (Valimex S.L., catalog number: 100M1006)
5. Plastic alveolus tray (Projar, catalog number: 4400000S28)
6. Cling film (KINGSWAY, catalog number: KING756046)
7. Aluminum foil (Alujet-Universal, catalog number: 293-4176)
8. Mortar and pestle (LGB, catalog number: MORN-075-001)
9. Tweezers (Clink, catalog number: FORS-003-002)
10. Micro spatula (Clink, catalog number: SPNS-150-005)
11. Centrifuge tubes with screw cap, 15 mL (Astik’s, catalog number: PTGP-E15-025)
12. Crimp neck vials ND11 (VWR, catalog number: 548-8008A)
13. Crimp caps with septa for crimp neck vials ND11 (VWR, catalog number: 548-8004A)
14. Minisart ^®^ syringe filters PTFE 0.2 μm (Sartorius, catalog number: 17573-ACK)
15. Gas chromatography column DB-5MS UI (Agilent, catalog number: 122-5562UI)

**Chemicals and solvents**

1. Sodium dodecyl sulfate (SDS) (PanReac AppliChem ITW Reagents, catalog number: A2263.0100)
2. Sodium hypochlorite (PanReac AppliChem ITW Reagents, catalog number: 212297.1211)
3. Methanol (VWR, catalog number: 20864.32)
4. Mandelonitrile (Sigma-Aldrich, catalog number: 116025)
5. Ethanol absolute (VWR, catalog number: 20821.365)
6. Acetonitrile (VWR, catalog number: 83639.320)
7. 2,2,2-Trifluoro-N-methyl-N-(trimethylsilyl)acetamide (MSTFA) (Supelco, catalog number: 1.11805)
8. Silicone oil for oil baths (VWR, catalog number: 24610.363)
9. Autoclaved distilled water
10. *
Arabidopsis* seeds sterilization solution (see Recipes)

## Equipment

1. Single-channel pipettes PIPETMAN ^®^ L P1000 (Gilson, catalog number: FA10006M)
2. Single-channel pipettes PIPETMAN ^®^ L P200 (Gilson, catalog number: FA10005M)
3. Single-channel pipettes PIPETMAN ^®^ L P20 (Gilson, catalog number: FA10003M)
4. Single-channel pipettes PIPETMAN ^®^ L P2 (Gilson, catalog number: FA10001M)
5. Elmasonic S30H (Elma Schmidbauer GmbH, catalog number: 1001955)
6. Heating oven Binder E028 (BINDER GmbH, catalog number: 9010-0001)
7. Sorvall X4F Pro centrifuge (Thermo Scientific, catalog number: 75009026)
8. Digital rotary evaporator (VWR, catalog number: 531-1365P)
9. Precision balance LPW 2103i (VWR, catalog number: 611-3283)
10. Agilent 7890B gas chromatography system coupled to an Agilent 7010 triple quadrupole mass spectrometer (Agilent)
11. Freezer -80 °C
12. Sanyo MLR-350-H Panasonic growth chamber
13. Laminar flow hood
14. 4 °C chamber

## Software

1. MassHunter Workstation software version B.07.01 (Agilent)
2. Microsoft office Excel
3. Statistical analysis software such as GraphPad Prism 9, OriginPro 9.0, or Statgraphics

## Procedure

- ***A. thaliana* seeds sterilization**
  1. Place approximately 80 seeds of *A. thaliana* Col-0 into a 1.5 mL tube.
  2. In a laminar flow hood, add 1 mL of 70% ethanol to the tubes and mix by inversion for 2 min.
  3. Remove ethanol using a micropipette, add 1 mL of *Arabidopsis* seeds sterilization solution (see Recipes) to the tube, and mix by inversion for 12 min.
  4. Remove the sterilization solution using a micropipette and perform five washing cycles with autoclaved distilled water.
  5. Leave the seeds in water until use (see Note 2).
- ***
A. thaliana* seed sowing
**
  1. Prepare a plastic seed tray with autoclaved peat moss: vermiculite (2:1) for 20 alveolus of 6 × 6 × 8 cm.
  2. Place five sterilized seeds per alveolus avoiding corners.
  3. Cover the planted seed tray with a plastic film and leave it at 4 °C for five days to synchronize seed germination.
  4. Place the seed tray (that has been stratified at 4 °C for five days) into the growth chamber at 23 ± 1 °C, >70% relative humidity, and a 16:8 h day/night photoperiod.
  5. Grow plants for 2–3 weeks (see Note 3).
- ***
A. thaliana* plants infestation
**
Infestation assay protocol with spider mite *T. urticae* was previously reported by Cazaux et al. (2014).
  1. Divide plants into two groups: one for control plants and the other one for infestation with *T. urticae.
*
  2. Perform the infestation. Place 20 adult female mites per plant and cover the rosette with a ventilated plastic cup to avoid mite scape. Leave in the chamber for 24 h at 25 ± 1 °C, > 70% relative humidity, and a 16:8 h day/night photoperiod.
  3. Remove the plastic cup carefully, cut the rosette, and quickly freeze it in liquid nitrogen.
  4. Store plant material at -80 °C until use.
- **
Mandelonitrile extraction
**
The mandelonitrile isolation protocol was adapted from Madala et al. (2014) as follows.
  1. Crush plant material until it reaches a fine powder using a mortar and pestle (use liquid nitrogen to prevent thawing of the material).
  2. Weigh the plant material using a precision balance (plant material ≥ 100 mg; see Note 4).
  3. In a small mortar, add 3 mL of methanol and the frozen plant material. Homogenate with a pestle and transfer the homogenate to a 15 mL Falcon tube (see Note 5).
  4. Sonicate for 20 min at room temperature (see Note 6).
  5. Incubate at 60 °C for 10 min using a heating oven.
  6. Centrifuge at 11,500× *g* for 5 min at room temperature.
  7. Collect the supernatants in a 10 mL flask (see Note 7) and remove the methanol on the rotary evaporator at 50 °C. Make sure that samples are completely dry (see Note 8).
- **
GC/MS analyses
**
  1. Derivatization by silylation with N-methyl-N-(trimethylsilyl) trifluoroacetamide (MSTFA):
    - Weigh 50 μL of mandelonitrile in a 10 mL flask to prepare the standard curve (see Note 7).
    - Add 100 μL of MSTFA to the mandelonitrile sample and to the Col-0 samples.
    - Mix well to achieve a homogeneous solution.
    - Incubate at 60 °C in an oil bath for 30 min protected from light (see Note 9).
    - Cool down the samples and the standard at room temperature.
  2. Samples and standard curve preparation for GC analyses:
    - Prepare standard solutions of mandelonitrile (derivatized with MSTFA) in acetonitrile solvent at 2.5, 5, 10, 25, 50, 100, 250, and 500 ng/μL concentrations using 5 mL vials.
    - Prepare samples by adding 900 μL of acetonitrile to the derivatized Col-0 samples.
    - Filter the samples and the standards through a 0.2 μm syringe filter and place the sample and the standards in crimp neck vials for GC analyses.
  3. GC analyses:
    - Inject 1 μL of the standard solution or the sample in a DB-5MS UI column of 60 m × 0.25 mm with 0.25 μm film thickness with a pressure of 17.7 psi, septum purge flow of 3 mL/min, and spitless time of 1.5 min (see Note 10).
    - Set the injector temperature to 250 °C.
    - Set the oven temperature to 60 °C, hold for 3 min, and then raise 10 °C/min up to 300 °C and maintain at 300 °C for 6 min.
    - Set the transfer line temperature to 280 °C.
    - Set the collision gas (nitrogen) and the quench gas (helium) with a flow rate of 1.5 mL/min and 2.25 mL/min, respectively.
    - Set the operation mode as electron ionization mode at 280 °C and 70 eV with multiple reaction monitoring (MRM) to monitor ion transitions (see Notes 11 and 12).

**Figure 2.**
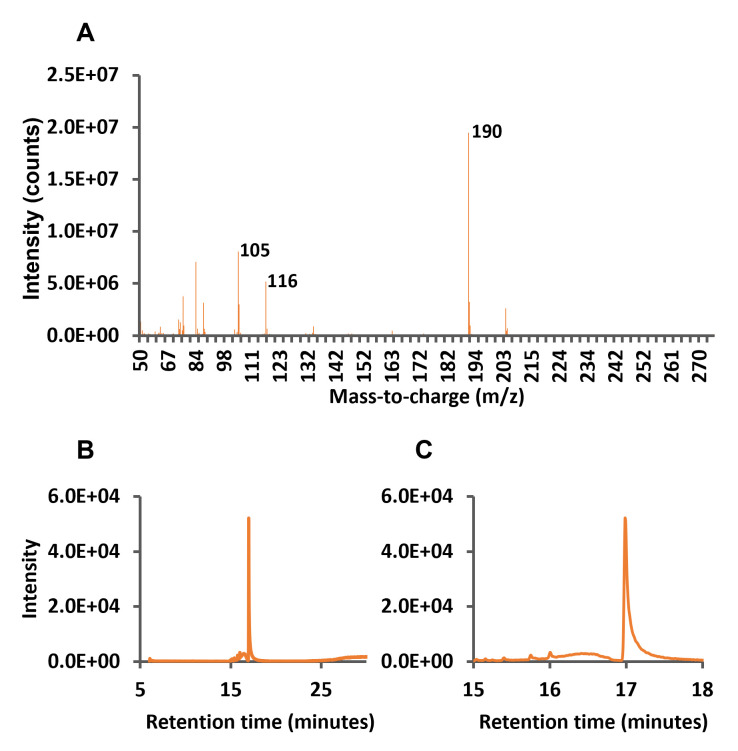
Full scan and total ion chromatograms (TICs) of mandelonitrile standard. A. Full scan chromatogram of mandelonitrile indicating the selected ion products. B. TIC of mandelonitrile at 25 ng/mL. C. Amplification of the retention times between 15 and 18 min.

The total ion and the retention time zoom chromatograms between minutes 15 and 18 are shown in Figure 2A and 2B, respectively.

## Data analysis

Three independent experiments of *A. thaliana* spider mite infestation were performed. Therefore, three independent mandelonitrile *A. thaliana* extractions, derivatizations, and quantifications were done, always including the standard molecule.

The product ion 190 ( *m/z* ) was designated as a quantifier ion; therefore, this product ion was selected for analyzing the standard and the samples. MassHunter Workstation software version B.07.01 was used to analyze the results and to export the data in csv format, to analyze using Excel software.

**Figure 3.**
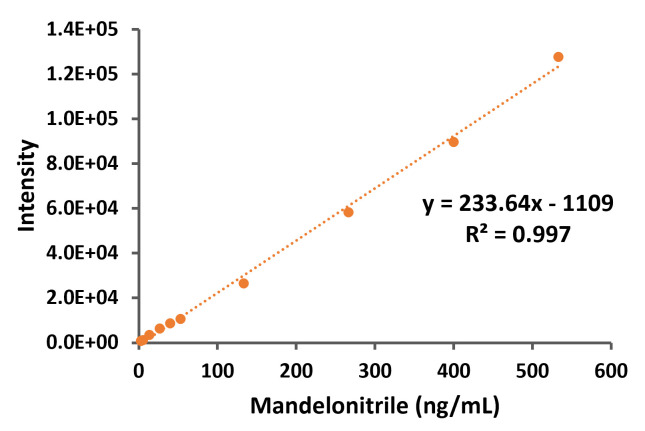
Standard curve of mandelonitrile derivatized

First, the standard curve was analyzed ( Figure 3 ) by graphical representation of the intensity obtained from the mass spectrometer (from the 190-product ion peaks) vs. the mandelonitrile concentration.

**Figure 4.**
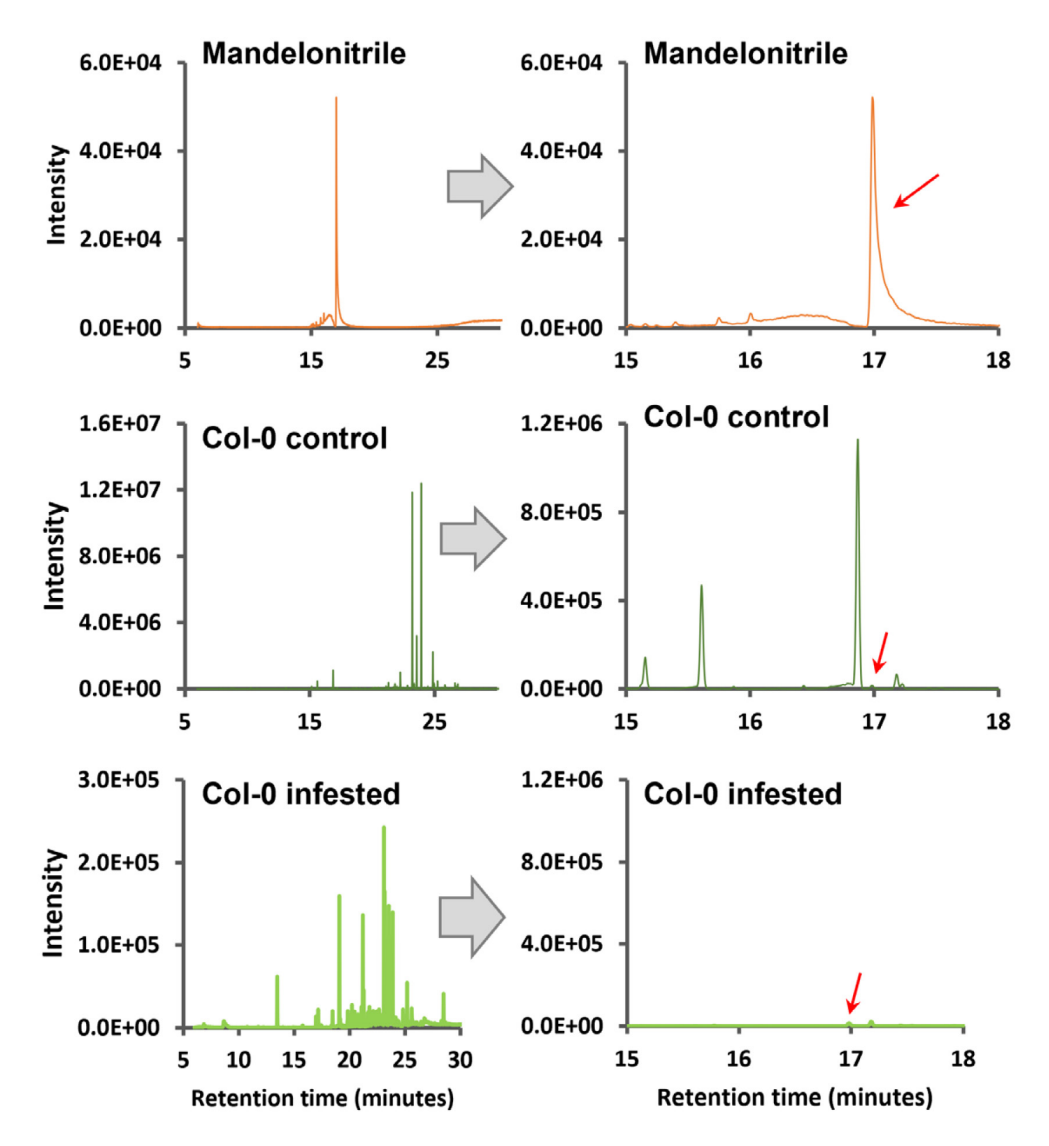
Total ion chromatograms of mandelonitrile and *Arabidopsis* Col-0 samples with their amplification zone of retention times between 15 and 18 min. Red arrows indicate the corresponding peak of mandelonitrile derivatized.

Next, the total ion chromatograms (TICs) of the samples were compared to the standard in order to find the derivatized mandelonitrile peak in the *Arabidopsis* samples. As shown in Figure 4 , the TICs from *Arabidopsis* Col-0 samples present different peaks, both in the control and in the mites-infested samples, including the mandelonitrile ones marked with a red arrow in each chromatogram.

**Figure 5.**
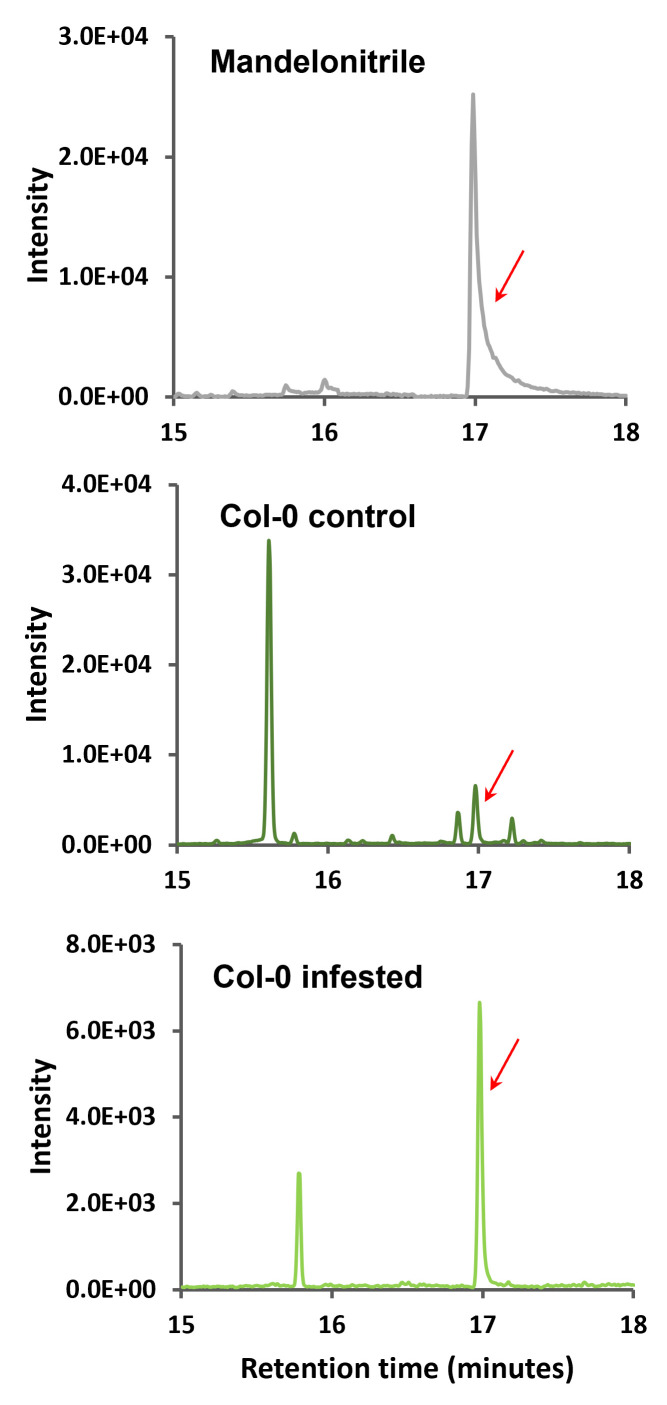
Multiple reaction monitoring (MRM) chromatograms of 205–105 *m/z* ion transition of mandelonitrile and *Arabidopsis* Col-0 samples. Retention time zoom between 15 and 18 min is shown, and red arrows indicate the corresponding peak of ion product 105 ( *m/z* ) of mandelonitrile derivatized.

**Figure 6.**
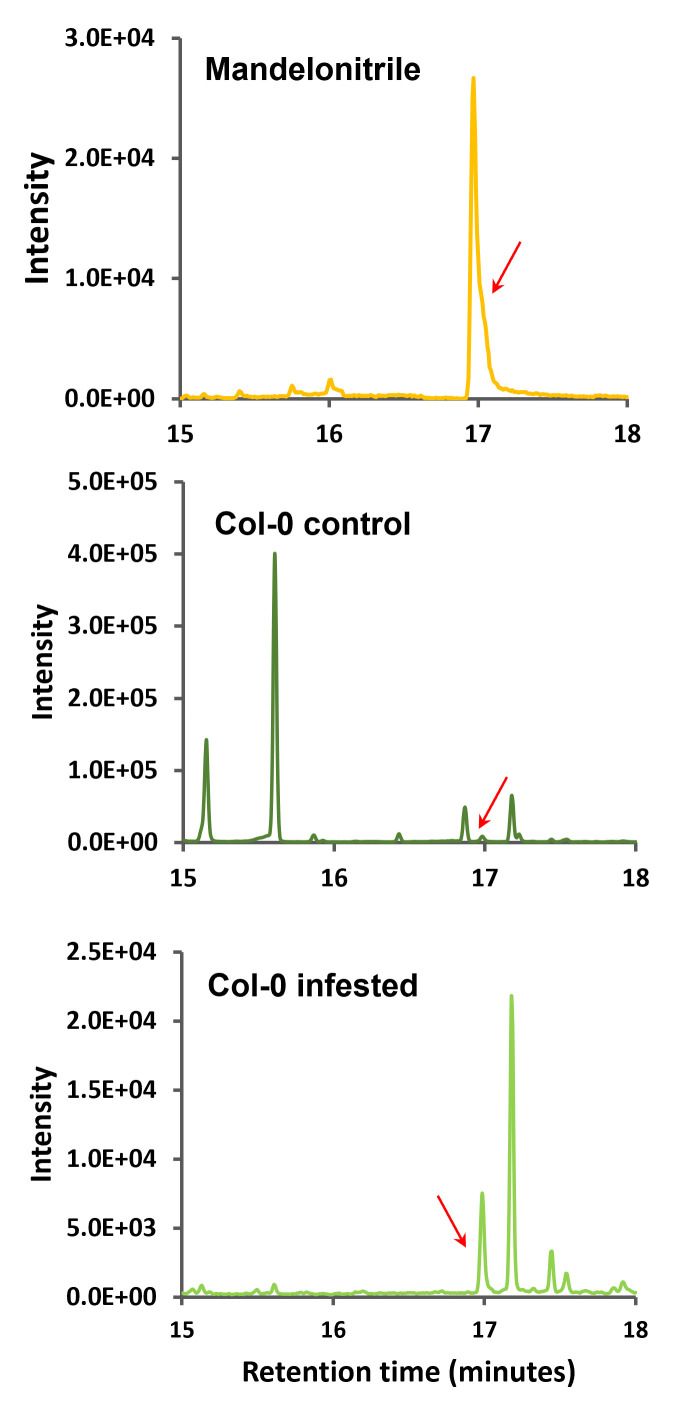
Multiple reaction monitoring (MRM) chromatograms of 205–190 *m/z* ion transition of mandelonitrile and *Arabidopsis* Col-0 samples. Retention time zoom between 15 and 18 min are shown, and red arrows indicate the corresponding peak of ion product 190 ( *m/z* ) of mandelonitrile derivatized.

Subsequently, the corresponding chromatograms of the product ion 105 ( *m/z* ) that was selected as the qualifier ion ( Figure 5 ), and the chromatograms of the product ion 190 ( *m/z* ) that was selected as quantifier ion ( Figure 6 ) were analyzed.

**Figure 7.**
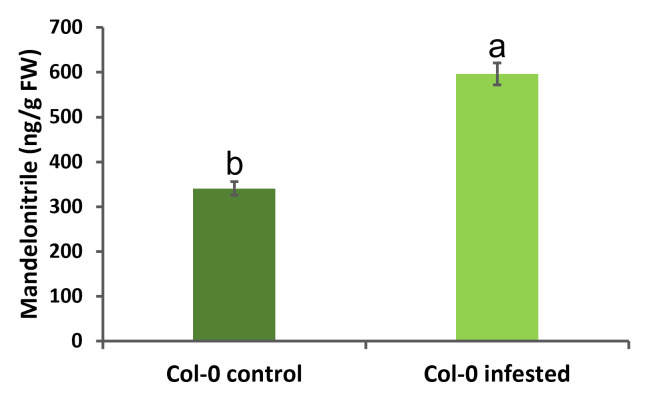
Mandelonitrile quantification in *A. thaliana* in control and infested with mites. Data are represented as nanograms of mandelonitrile/grams of fresh weight. Data are means ± SE of three biological replicates (p < 0.05, one-way ANOVA followed by Tukey’s test).

Finally, the mandelonitrile concentration data in the *A. thaliana* samples were obtained and represented ( Figure 7 ). Normality and homoscedasticity of data were analyzed using OriginPro 9.0 software. When the data fulfilled both assumptions, one-way ANOVA followed by Tukey’s test were run.

## Notes

1. *
T. urticae* colony is reared on beans ( *Phaseolus vulgaris* ) in a growth chamber at 25 °C ± 1 °C, > 70% relative humidity, and with a 16:8 h day/night photoperiod.
2. Do not leave seeds in water for more than 60 min before sowing. It is better to program the sowing to do it as soon as seed sterilization is completed.
3. Keep an eye on seed germination every two days. When seeds start germinating, perform holes in the plastic film to allow plants to acclimate to the growth chamber environment. Two or three days after making the holes, remove the plastic film. Water the trays every 2–3 days with 15 mL per alveolus (it is important to ensure that there is no humidity excess that causes condensation on the underside of the leaves). When plants have grown, remove them from each alveolus with the help of tweezers, to leave only one plant per alveolus (preferably the most centrally located plant).
4. Usually, with 10 plants, approximately 200 mg of material is obtained. It is recommended to use 100–300 mg of plant material for the mandelonitrile extraction step.
5. It is not necessary to use 15 mL Falcon tubes; other containers, such as glass flasks, can be used.
6. The ultrasonic frequency of the sonicator is 37 kHz. This frequency is a preset ultrasonic frequency of the sonicator model used.
7. 10 mL flasks were used, but smaller vials can also be used taking into account the availability of adapters for the rotary evaporator.
8. Samples have an oily appearance.
9. The use of an oil bath is not mandatory; other options can be used (water bath, incubation chamber, oven) while the sample is incubated at 60 °C. MSTFA is light-sensitive. Therefore, derivatization conditions were established using mandelonitrile; assessing if derivatization was complete was done by nuclear magnetic resonance.
10. It is recommended to include samples with only solvent (acetonitrile) between the standard and the samples to be analyzed to ensure that the system is thoroughly cleaned, in case there are any remains of the retained compound.
It is not necessary to use a 60 m column; shorter columns (25–30 m) can also be used. Just keep in mind that if the length of the column is reduced, it may be necessary to increase the total run time.
11. The precursor ion presents an *m/z* of 205. The ion transitions selected as quantifier and qualifier were the product ions with an *m/z* of 190 and 105, respectively. The collision energy of the product ions was 5 eV and 20 eV for the 190- and 105-product ion, respectively. The product ion with an *m/z* of 116 was also identified in the analyses. Still, since it has a very low peak intensity, it was not used as a quantifier or a qualifier.
It is important to analyze the three ions at the same time (triple quad) to rule out false positives or negatives. If one of the three ions does not come out right in a sample, that sample will have to be repeated.
12. Method validation was performed by Arnaiz et al. (2022), with a limit of detection and quantification of 5 ± 1 ng/mL and 12 ± 2 ng/mL, respectively.

## Recipes

1. ***
Arabidopsis* seeds sterilization solution
**
1% SDS, 5% sodium hypochlorite

## References

[1] Andexer J. , von Langermann J. , Mell A. , Bocola M. , Kragl U. , Eggert T. and Pohl M. ( 2007 ). An R-selective hydroxynitrile lyase from * Arabidopsis thaliana * with an alpha/beta-hydrolase fold . Angew Chem Int Ed Engl 46 ( 45 ): 8679 - 8681 . 17907254 10.1002/anie.200701455

[2] Arnaiz A. , Santamaria M. E. , Rosa-Diaz I. , Garcia I. , Dixit S. , Vallejos S. , Gotor C. , Martinez M. , Grbic V. and Diaz I. ( 2022 ). Hydroxynitrile lyase defends * Arabidopsis * against * Tetranychus urticae * . Plant Physiol 189 ( 4 ): 2244 - 2258 . 35474139 10.1093/plphys/kiac170 PMC9342993

[3] Beran F. , Köllner T. G. , Gershenzon J. and Tholl D. ( 2019 ). Chemical convergence between plants and insects: biosynthetic origins and functions of common secondary metabolites . New Phytol 223 ( 1 ): 52 - 67 . 30707438 10.1111/nph.15718

[4] Bernal-Vicente A. , Petri C. , Hernández J. A. and Diaz-Vivancos P. ( 2020 ). Biochemical study of the effect of stress conditions on the mandelonitrile-associated salicylic acid biosynthesis in peach . Plant Biol(Stuttg) 22 ( 2 ): 277 - 286 . 31674699 10.1111/plb.13066

[5] Cazaux M. , Navarro M. , Bruinsma K. A. , Zhurov V. , Negrave T. , Van Leeuwen T. , Grbic V. and Grbic M. ( 2014 ). Application of two-spotted spider mite * Tetranychus urticae * for plant-pest interaction studies . J Vis Exp 4 ( 89 ): 51738 . 10.3791/51738 PMC4211727 25046103

[6] Chandra A. , and Nair M. G. ( 1993 ). Quantification of benzaldehyde and its precursors in Montmorency cherry( * Prunus cerasus * L.) kernels . Phytochem Anal 4 ( 3 ): 120 - 123 .

[7] Chassagne D. , Crouzet J. C. , Bayonove C. L. and Baumes R. L. ( 1996 ). Identification and quantification of passion fruit cyanogenic glycosides . J Agric Food Chem 44 ( 12 ): 3817 - 3820 .

[8] Flematti G. R. , Waters M. T. , Scaffidi A. , Merritt D. J. , Ghisalberti E. L. , Dixon K. W. and Smith S. M. ( 2013 ). Karrikin and cyanohydrin smoke signals provide clues to new endogenous plant signaling compounds . Mol Plant 6 ( 1 ): 29 - 37 . 23180672 10.1093/mp/sss132

[9] Gleadow R. M. , and Møller B. L. ( 2014 ). Cyanogenic glycosides: Synthesis, physiology, and phenotypic plasticity . Annu Rev Plant Biol 65 : 155 - 185 . 24579992 10.1146/annurev-arplant-050213-040027

[10] Hirvi T. , and Honkanen E. ( 1985 ). Analysis of the volatile constituents of black chokeberry( * Aronia melanocarpa * Ell.) . J Sci Food Agric 36 ( 9 ): 808 - 810 .

[11] Kadow D. , Voss K. , Selmar D. and Lieberei R. ( 2012 ). The cyanogenic syndrome in rubber tree * Hevea brasiliensis * : tissue-damage-dependent activation of linamarase and hydroxynitrile lyase accelerates hydrogen cyanide release . Ann Bot 109 ( 7 ): 1253 - 1262 . 22451599 10.1093/aob/mcs057 PMC3359917

[12] Madala N. E. , Steenkamp P. A. , Piater L. A. and Dubery I. A. ( 2014 ). Metabolomic insights into the bioconversion of isonitrosoacetophenone in * Arabidopsis thaliana * and its effects on defense-related pathways . Plant Physiol Biochem 84 : 87 - 95 . 25240267 10.1016/j.plaphy.2014.08.023

[13] Møller B. L. ( 2010 ). Functional diversifications of cyanogenic glucosides . Curr Opin Plant Biol 13 ( 3 ): 338 - 347 . 20197238 10.1016/j.pbi.2010.01.009

[14] Neilson E. H. , Goodger J. Q. D. , Woodrow I. E. and Møller B. L. ( 2013 ). Plant chemical defence: at what cost ? Trends Plant Sci 18 ( 5 ): 250 - 258 . 23415056 10.1016/j.tplants.2013.01.001

[15] Park D. S. , Grodnitzky J. A. and Coats J. R. ( 2002 ). QSAR evaluation of cyanohydrins’ fumigation toxicity to house fly( * Musca domestica * ) and lesser grain borer( * Rhyzopertha dominica * ) . J Agric Food Chem 50 ( 20 ): 5617 - 5620 . 12236687 10.1021/jf020361t

[16] Pastorczyk M. , Kosaka A. , Piślewska-Bednarek M. , López G. , Frerigmann H. , Kułak K. , Glawischnig E. , Molina A. , Takano Y. and Bednarek P. ( 2020 ). The role of CYP71A12 monooxygenase in pathogen-triggered tryptophan metabolism and * Arabidopsis * immunity . New Phytol 225 ( 1 ): 400 - 412 . 31411742 10.1111/nph.16118

[17] Rajniak J. , Barco B. , Clay N. K. and Sattely E. S. ( 2015 ). A new cyanogenic metabolite in * Arabidopsis * required for inducible pathogen defense . Nature 525 ( 7569 ): 376 - 379 . 26352477 10.1038/nature14907 PMC4629851

[18] Zagrobelny M. and Møller B. L. ( 2011 ). Cyanogenic glucosides in the biological warfare between plants and insects: the Burnet moth-birdsfoot trefoil model system . Phytochemistry 72 ( 13 ): 1585 - 1592 . 21429539 10.1016/j.phytochem.2011.02.023

[19] Zagrobelny M. , Olsen C. E. , Pentzold S. , Fürstenberg-Hägg J. , Jørgensen K. , Bak S. , Møller B. L. and Motawia M. S. ( 2014 ). Sequestration, tissue distribution and developmental transmission of cyanogenic glucosides in a specialist insect herbivore . Insect Biochem Mol Biol 44 : 44 - 53 . 24269868 10.1016/j.ibmb.2013.11.003

